# The Effect of Platelet-Rich Plasma on the Intra-Articular Microenvironment in Knee Osteoarthritis

**DOI:** 10.3390/ijms22115492

**Published:** 2021-05-23

**Authors:** Dawid Szwedowski, Joanna Szczepanek, Łukasz Paczesny, Jan Zabrzyński, Maciej Gagat, Ali Mobasheri, Sławomir Jeka

**Affiliations:** 1 Orthopedic Arthroscopic Surgery International (O.A.S.I.) Bioresearch Foundation, Gobbi N.P.O., 20133 Milan, Italy; 2 Department of Orthopaedics and Trauma Surgery, Provincial Polyclinical Hospital, 87100 Torun, Poland; 3 Centre for Modern Interdisciplinary Technologies, Nicolaus Copernicus University, 87100 Torun, Poland; szczepanekj@umk.pl; 4 Orvit Clinic, Citomed Healthcare Center, 87100 Torun, Poland; drpaczesny@gmail.com (Ł.P.); zabrzynski@gmail.com (J.Z.); 5 Department of Histology and Embryology, Faculty of Medicine, Collegium Medicum in Bydgoszcz, Nicolaus Copernicus University in Torun, 85067 Bydgoszcz, Poland; mgagat@cm.umk.pl; 6 Research Unit of Medical Imaging, Physics and Technology, Faculty of Medicine, University of Oulu, FI-90014 Oulu, Finland; ali.mobasheri@oulu.fi; 7 Department of Regenerative Medicine, State Research Institute Centre for Innovative Medicine, Santariskiu 5, LT-08406 Vilnius, Lithuania; 8 Departments of Orthopedics, Rheumatology and Clinical Immunology, University Medical Center Utrecht, 3508 GA Utrecht, The Netherlands; 9 Department of Joint Surgery, The First Affiliated Hospital, Sun Yat-sen University, Guangzhou 510080, China; 10 Department and Clinic of Rheumatology and Connective Tissue Diseases, University Hospital No. 2, Collegium Medicum UMK, 85168 Bydgoszcz, Poland; s.jeka@wp.pl

**Keywords:** platelet-rich plasma (PRP), knee osteoarthritis (KOA), cartilage repair, regenerative medicine

## Abstract

Knee osteoarthritis (KOA) represents a clinical challenge due to poor potential for spontaneous healing of cartilage lesions. Several treatment options are available for KOA, including oral nonsteroidal anti-inflammatory drugs, physical therapy, braces, activity modification, and finally operative treatment. Intra-articular (IA) injections are usually used when the non-operative treatment is not effective, and when the surgery is not yet indicated. More and more studies suggesting that IA injections are as or even more efficient and safe than NSAIDs. Recently, research to improve intra-articular homeostasis has focused on biologic adjuncts, such as platelet-rich plasma (PRP). The catabolic and inflammatory intra-articular processes that exists in knee osteoarthritis (KOA) may be influenced by the administration of PRP and its derivatives. PRP can induce a regenerative response and lead to the improvement of metabolic functions of damaged structures. However, the positive effect on chondrogenesis and proliferation of mesenchymal stem cells (MSC) is still highly controversial. Recommendations from in vitro and animal research often lead to different clinical outcomes because it is difficult to translate non-clinical study outcomes and methodology recommendations to human clinical treatment protocols. In recent years, significant progress has been made in understanding the mechanism of PRP action. In this review, we will discuss mechanisms related to inflammation and chondrogenesis in cartilage repair and regenerative processes after PRP administration in in vitro and animal studies. Furthermore, we review clinical trials of PRP efficiency in changing the OA biomarkers in knee joint.

## 1. Introduction

Platelet-rich plasma is an autologous solution of highly concentrated platelets dispersed in a small capacity of plasma. Enthusiasm for the therapeutic potential of platelets is based on its rich complement of anabolic growth factors and anti-inflammatory cytokines in the platelets, which induce cellular proliferation, migration, differentiation, angiogenesis, and extracellular matrix (ECM) synthesis [1,2]. In addition, the functional mechanisms of PRP in OA treatment have been explained by its effect on modulating critical pro-inflammatory mediators and catabolic enzymes, as well as maintaining joint homeostasis [3,4]. It has been shown to have a positive effect on tissue healing is observed with a supply of platelets of at least 1,000,000/µL in 5 mL of plasma [5]. Platelets contains three types of granules: α-granules, dense granules, and lysosomal granules. Alpha-granules are a source of growth factors, including platelet-derived growth factors (PDGF), insulin-like growth factor-1 (IGF-1), vascular endothelial growth factor (VEGF), transforming growth factor (TGF). The overall functions of these specific growth factors released by PRP are discussed in Table 1.

The major role of growth factors contained in PRP is to recruit and activate other immune cells or induce endothelial cell inflammation. Additional factors included in α-granules are chemokines and cytokines, such as platelet factor 4 (PF4), pro-platelet basic protein, and P-selectin, which are involved in stimulating cell chemotaxis, proliferation and maturation, as well as modulating inflammatory molecules [21]. The dense granules contain ADP, ATP, serotonin, dopamine, polyphosphates, histamine, and epinephrine that modifies platelet activation and thrombus formation. Most importantly, many of these elements have immune cell-modifying effects [22]. Histamine and serotonin increase the permeability of capillaries, enabling the migration of cells involved in the inflammatory process, such as macrophages [23]. As a result, fibroblasts, mesenchymal stem cells and autologous chondrocytes are stimulated. Lysosomal granules contain hydrolases, cathepsin D and E, elastase and lysozyme, and many other proteins whose physiological role has not been well characterized so far [24].

Intra-articular injections with PRP have been used with increasing frequency in the treatment of musculoskeletal pathologies, such as chronic sports-related injuries of the muscles and tendons, and degenerative joint diseases. More recently, several clinical studies showed significant improvement with PRP treatment for OA compared with hyaluronic acid injection and placebo [1]. Despite the increased interest in PRP use for the treatment of OA, the precise mechanisms and effects of PRP on knee joint tissues remain unclear. Substantial variations in baseline platelet counts among individual patients [25] as well as differences between PRP preparation methods [26] may prevent the control of the correlation between platelet dose and concentration as well as released bioactive growth factors concentration.

**Figure 1:**
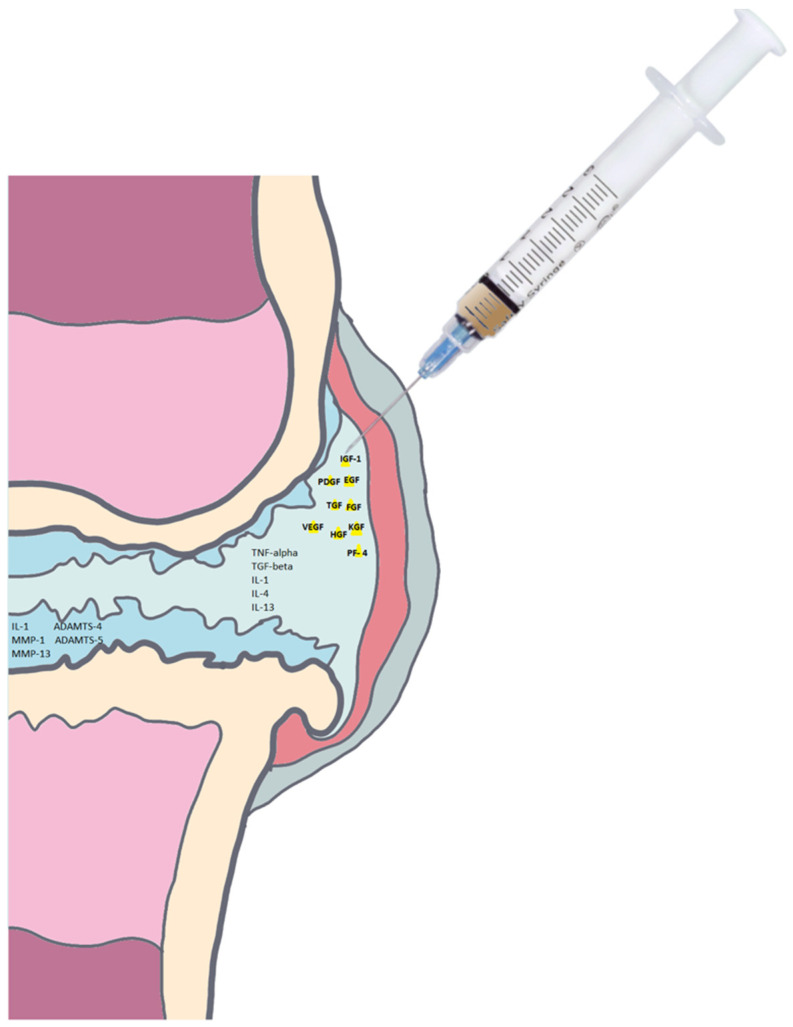
Growth factors (yellow triangles) released in the OA knee joint after PRP injection. TNF-tumor necrosis factor, IGF-insulin-like growth factor, TGF-transforming growth factor. VEGF-vascular endothelial growth factor, ADAMTS-a disintegrin and metalloproteinase with thrombospondin-like motifs, IL-interleukin, MMP-matrix metalloproteinase, EGF-epidermal growth factor, HGF-hepatocyte growth factor, FGF-fibroblast growth factor, KGF-keratinocyte growth factor, PF-4-platelet factor 4.

It is assumed that cartilage damage leads to inflammation and inflammatory component play a significant role in the development and progression of OA. Interleukin-1β (IL-1β) and tumor necrosis factor-α (TNF-α) are the catabolic cytokines in the OA knee joint which cause the activity and levels of matrix metalloproteinases (MMP) and elastases to increase by both synovial cells and chondrocytes. Another factor associated with OA progression are epigenetic changes in articular chondrocytes [27]. Modifications to the epigenetic pattern can lead to genetic disruptions that result in the overexpression of cartilage-degrading proteases and inflammatory process factors. Responsible for the breakdown of the articular extracellular matrix (ECM), enzymes may trigger osteoarthritis (OA) progression and inhibit regenerative tissue production during cartilage repair [28] (Figure 1). The catabolic and inflammatory intra-articular environment that exists in osteoarthritis may be influenced by the administration of PRP. Consequently, the molecular pathways of PRP treatment efficiency in knee OA have been the point of focus in clinical studies.

## 2. In Vitro Studies

The effect of PRP on chondrogenesis and ECM turnover is still controversial. Although previous studies demonstrated that PRP can enhance chondrogenesis and as well as inhibit inflammatory responses in cartilaginous tissue [29,30], the study by Rikkers et al. [31] proposed that PRP does not exert anti-inflammatory effects on chondrocytes in an in vitro inflammation model. Additionally, they demonstrated that production of cartilage ECM was strongly down-regulated by PRP. Furthermore, although PRP stimulated chondrocyte proliferation, it was shown to have no effect on the migration of chondrocytes. However, Kruger et al. [32] demonstrated using in vitro models that human PRR (number of platelets was 0.6–1.3 × 10^10^ and leukocytes were <0.3 × 10^4^ mL) can stimulate the migration and the chondrogenic differentiation of human subchondral mesenchymal progenitor cells. Although osteogenic or adipogenic differentiation was not evident, staining of proteoglycans and type II collagen indicated that PRP effectively induced the formation of cartilage matrix. Another study by Chen et al. [33] demonstrated that PRP (the platelet and leukocyte counts were 339.3 × 10^3^/µL and 0.821 × 10^3^/µL, respectively) can efficiently suppress the adipogenesis and inflammatory stimulus of infrapatellar fat pad (IFP) adipocytes. The IFP and the adipocytes within it may participate in the pathophysiology of knee OA by releasing various proinflammatory adipokines that can contribute to dedifferentiation and inflammation in chondrocytes [34,35,36]. Moreover, adiponectin and leptin can further promote cartilage degradation by inducing the infiltration of leukocytes and monocytes in OA cartilage [37,38,39]. This study showed that the combination of PRP and hyaluronic acid (HA) decreased the inflammatory potential of IFP adipocytes through the down-regulation of proinflammatory cytokines and adipokines. Realistically and pragmatically, IFP could be a potential therapeutic target for knee OA, and the administration of PRP can efficiently suppress the adipocytokine-mediated inflammatory process and, therefore stimulate the formation of neocartilage. Other scholars who reported new aspects of the anti-inflammatory impact of activated PRP are Bendinelli et al. [40]. Their research results have shown that thrombin-activated PRP containing not only reparative growth factors, but also hepatocyte growth factor (HGF), together with IL-4, TNF-α and TGF-β1, might justify why it should be applied in articular cartilage regeneration. Their finding that CXCR4 expression diminished in the chondrocytes exposed to activated PRP may be clinically significant in inflamed synovium owing to the reduction of the secretion of matrix metalloproteinase (MMP). Using a human cartilage coculture model and synovial tissue from OA patients, O’Brien et al. conducted a comparison of PRP anti-inflammatory effects with amniotic viscous fluid. As a result, both biologic treatments contributed to considerable reductions of disintegrin and metalloproteinase with thrombospondin motifs-5 (ADAMTS-5) and tissue inhibitor of metalloproteinases (TIMP-1) gene expression in cartilage and synovium for a maximum of 72 h. Still, it was demonstrated that unlike PRP, amnion did not have any influence on the concentration of nitric oxide at any time point. Although PRP had mostly similar anti-inflammatory gene expression to that of amniotic fluid, the combination of these treatments did not demonstrate any additive effects in the reduction of the inflammatory process. 

One of the main problems with PRP is the lack of standardization. The large number and variability of commercially available PRP systems lead to lack of consistency among studies. These devices vary in PRP collection volumes and preparation protocols. It has been shown that very high concentrations of growth factors are not necessarily advantageous for cell stimulatory processes and might be not effective due to the limited quantity of cell membrane receptors [41]. Additionally, it is important to consider that many growth factors in PRP have a limited biological half-life, which can explain, at least in part, the variability seen with PRP treatment outcomes. Once the growth factor levels are too high compared to the available receptors, they might negatively affect cell function [22,42]. The problem with PRP standardization could be solved using characterized lyophilized PRP powder. Hahn et al. [43] evaluated the effects of PRP powder with characterized growth factors, comprising VEGF (379.3 pg/mL), TGF-β1 (78,048.0 pg/mL), bFGF (30.1 pg/mL), PDGF (3395.8 pg/mL), and IGF-1 (591.9 pg/mL), on human chondrocytes derived from hyaline articular cartilage. They investigated the impact of varying PRP doses on different concentrations, timing, and number of applications to human chondrocytes. Contrary to the above-mentioned studies, their research demonstrated primarily that PRP concentration and stimulation frequency had a direct influence on chondrocyte proliferation and metabolic activity in a dose-dependent manner. Thus, it is suggested that future basic science and clinical studies should more often consider the frequency of PRP injections application and the short biological half-lives of the growth factors therein.

The therapeutic effects of intra-articular PRP treatment is thought to be mediated not only by a protective effect against the cytotoxicity of reactive oxygen species (ROS) and an anti-inflammatory effect but also by biomechanical modulating properties of PRP. The study by Sakata et al. [44] demonstrated that PRP enhanced superficial zone protein (SZP) secretion from synovium- and cartilage-derived cell and the presence of endogenous SZP in PRP. The lubrication properties of PRP from healthy volunteers on bovine articular cartilage showed decreased friction after PRP treatment compared with saline and hyaluronan. The friction coefficient of the cartilage with PRP was similar to that of synovial fluid. In addition, PRP stimulated proliferation in cells derived from articular cartilage, synovium, and the anterior cruciate ligament (ACL) isolated from patients undergoing ACL reconstruction.

The popularity of in vitro PRP research stems from the possibility of controlling the different parameters in these studies in a precise manner and achieving study results rapidly. Given that in vitro culturing may not reflect homeostatic conditions in vivo, the results of in vitro studies hardly translate into clinical practice. This may lead to the reporting of contradictory results between in vitro research data and the outcomes of clinical studies.

## 3. Animal Studies

The unresolved issues with the frequency of PRP administration in early OA have been studied in animal models. A study conducted by Chouhan et al. [45] was designed to investigate whether three injections of allogenic, leukocyte-poor PRP are superior to a single injection and to determine their time-dependent efficacy in a guinea pig model. Articular cartilage degeneration and synovial inflammation were evaluated according to OARSI recommendations [46]. They concluded thatin the short termsimilar anti-inflammatory effects can be observed after single as well as multiple PRP injections. Nevertheless, multiple PRP injections caused better long-term reduction in inflammation and only they produced the chondroprotective effect. By the same token, Khatab el al. [47] demonstrated in their study that multiple intra-articular PRP injections diminish synovial inflammation and can exert a protective effect on cartilage while alleviating pain in a collagenase-induced OA mouse model. Mice received three intra-articular injections of human, leukocyte-poor PRP or saline in the affected knee and were euthanized after three weeks so that cartilage damage and synovial inflammation on histological evaluation could be assessed. Moreover, the scholars used antibodies against iNOS, CD163, and CD206 in order to define various macrophages subtypes in the synovial membrane. Compared to saline-injected knees, PRP-injected knees featured a thinner synovial membrane, experienced less pain, and tended to show less cartilage damage in the lateral joint compartment. Kanwat et al. [48] showed that allogenic PRP injection decreased synovial vascularity in OA knees in Dunkin-Hartley guinea pig model. In addition, mean synovial fluid concentration of cartilage oligomeric matrix protein (COMP), one of the chondral damage markers, was lower in OA knees treated with PRP. The efficacy of PRP was examined not only in cartilage, synovium and synovial fluid, but also in meniscal mechanisms under normal and post-traumatic inflammatory conditions [49]. Reproducible defect on the meniscus was used to implant fibrin glue or PRP in New Zealand white rabbits. This study showed that PRP treatment results in an increase of catabolic molecules, especially those related to IL-1α-induced inflammation, and that it may accelerates fibrosis, instead of meniscal cartilage.

Another problem often associated with PRP that is the content of leukocytes in PRP. In a rat arthritis model, Araya et al. [50] showed that intra-articular injection of pure PRP was the most effective treatment for inhibition of the progression of synovitis and reduction of pain. They compared pure PRP, leukocyte-pure PRP and leukocyte-rich PRP to clarify the optimal PRP formulation. Knee arthritis was induced with high-dose monosodium iodoacetate intra-articular injection and IFP structural changes, and cartilage degeneration were assessed in the short term follow-up (5 and 14 days). Previous studies also suggested that structural changes in the IFP after persistent inflammation play an important role in residual pain in a rat arthritis model [51,52]. Hence, the progression of changes in the synovium and IFP can be suppressed and pain can be alleviated to a larger extent if pure PRP is administered early. Moreover, calcitonin gene-related peptide (CGRP) density in the IFP was evaluated to conduct an analysis of the mechanism of pain relief through the administration of PRP. Expressed in sensory nerves, this pain-related neuropeptide was considerably smaller in knees injected with pure PRP, which implies that it might one of the mechanisms for inhibiting pain sensitization. 

## 4. Human Clinical Trials

It is clear that in vitro and preclinical animal models are not ideal for the successful translation of many regenerative studies into widely adopted treatment guidelines and routine clinical practice. Fortunately, there is an increase in the understanding of the molecular functions of PRP treatment that affect clinical outcomes in knee OA. In 2016, Cole et al. [53] presented the results of the study that compared the effects of PRP to HA in patients with mild to moderate OA using an analysis of synovial fluid proinflammatory and anti-inflammatory markers and clinical outcome measures. The low-leukocyte autologous conditioned plasma (ACP) system (Arthrex, Naples, FL, USA was used in this study. Aspirated synovial fluid was analyzed using ELISA, in duplicate with the mean reported, for such catabolic factors as TNF-α, IL-1B/IL-F2, IL-1ra/IL-1F3, IL-6, and CXCL8/IL-8. It was concluded that the Western Ontario and McMaster Universities Osteoarthritis Index (WOMAC) pain score, which was the major clinical outcome measure, was not significant between the PRP and HA groups at any time point. Lastly, two proinflammatory cytokines—IL-1β and TNF-α—tended to decrease preceding a major difference in subjective outcomes favoring PRP. As indicated by this finding, PRP anti-inflammatory properties can be conducive to an improvement in OA symptoms.

Lana et al. [54] opposed the use of leukocyte-pure PRP in knee OA treatment. They suggested that particular leukocytes, such as neutrophils, release both pro and anti-inflammatory molecules and, when combined with activated platelets, their therapeutic potential increases. They play an important role in the inflammatory process preceding tissue regeneration due to their release of both pro- and anti-inflammatory molecules. In addition, plasticity of monocytes is important for the non-inflammatory role in tissue regeneration [55]. Providing a proteomic characterization of the synovial fluid and blood samples obtained from knee OA-treated subjects and juxtaposing the results with HA treatment Mariani et al. [56] conducted an analysis of both local and systemic effects induced by leukocyte-rich PRP. The plasma and the synovial fluid were tested for the presence of pro- and anti-inflammatory cytokines (IL-1β, IL-4, IL-6, IL-8, IL-10, IL-13, IL-17) and growth factors (b-FGF, HGF, PDGF-AB/BB). Eventually, irrespective of the time points analyzed, leukocyte-rich PRP injections modulated significant changes of cytokine concentrations in neither the synovial fluid nor the plasma. These outcomes differ from the evidence presented in in vitro studies in which the presence of leukocytes seems to induce a cellular pro-inflammatory response.

Another issue regarding PRP treatment in knee OA is whether using PRP alone or in combination with HA [57]. Xu et al. [58] published randomized clinical trial results comparing PRP combined with HA (PRP+HA) to PRP alone and HA alone for the treatment of mild to moderate knee OA. In their study, ELISA were used to quantify IL-1β, TNF-α, MMP-3, and TIMP-1 levels in synovial fluid. In addition, the synovium and cartilage were observed by high-frequency color Doppler. At 24 months, PRP+HA was more effective than PRP or HA alone in pain reduction and function improvement. Moreover, PRP+HA inhibited synovial inflammation. After 6 and 12 months, the levels of IL-1β, TNF-α, MMP-3, and TIMP-1 in the PRP and PRP+HA groups decreased. As a result, the combination of PRP and HA resulted in improved clinical outcomes for the treatment of OA than either PRP or HA alone.

By the same token, it was suggested that intra-articular PRP injections associated with pes anserinus PRP injections should be a treatment option for elderly patients with knee OA induced positive proteomic changes in the synovial fluid along with functional improvement [59].

The Osteoarthritis Research Society International (OARSI) and the International Cartilage Regeneration and Joint Preservation Society (ICRS) have published papers to highlight the importance of identifying and clinically validating structural (i.e., anatomical) and biochemical biomarkers to identify OA early and guide enhanced and more targeted treatments [60,61]. Several pathophysiological mechanisms of knee OA have been researched extensively [62,63]. The differences in clinical outcomes between the patients might arise from the differences in PRP composition, and in cartilage biology of treated subjects [64]. Consequently, to develop better guidelines of PRP treatment, outcome measures should be tailored to the clinical and biochemical phenotypes of OA. Advantages and disadvantages of PRP therapy are presented in Table 2. 

## 5. Conclusions

Research into the pathogenesis of OA has led to the development of a variety of products based on biologic factors that promote healing. The use of the autologous PRP is a rapidly growing field of orthopedics. Despite their wide clinical use, some of these products have been studied without rigorous scientific standards. The major challenges in knee OA treatment with PRP intra-articular injections is to understand the mechanism of action in the joint to optimize and standardize PRP formulations, identify the most suitable biomarkers for assessing treatment efficacy and reveal the underlying mechanisms involved in OA pathophysiology. Various experimental and clinical studies conducted to date have used either multiple injections or single injections and were able to demonstrate the positive effect of PRP on structural modulation and anti-inflammatory effects in the knee joint. The dosing schedule and the variations in the PRP formulations are also often debated due the large number and variability of commercially available PRP systems. Progress has been made in understanding the effectiveness of PRP on intra-articular homeostasis. However, further research is needed to develop a clearer mechanistic understanding and a widely adopted consensus regarding standardization of PRP preparations, which will to contribute to substantial tissue repair mechanisms and better clinical outcomes.

## Figures and Tables

**Table 1:** Growth factors in platelet and their source and function.

Growth Factor	Function	Source Cells	References
epidermal growth factor (EGF)	stimulates the growth, proliferation, and differentiation of mesenchymal and epithelial cells and mitogenesis of chondrocytes and osteoblasts; affects the synthesis and metabolism of the extracellular matrix; promotes angiogenesis; promotes the healing of wounds; enhances the effect of other growth factors; stimulates endothelial chemotaxis and angiogenesis; regulates the secretion of collagenase.	platelets, macrophages, monocytes	[6,7,8]
platelet-derived growth factor (PDGF)	stimulates chemotaxis and mitogenesis of fibroblasts, glial cells, and smooth muscles; increases the synthesis and secretion of collagen, as well as regulates the secretion of collagenase; activates the chemotaxis of fibroblasts, macrophages, and neutrophils; regulates the proliferation of bone cells activates TGF-b and stimulates chemotaxis toward the PDGF gradient.	platelets, endothelial cells, macrophages, monocytes, smooth muscle cells, osteoblasts	[8,9,10,11,12,13,14]
transformative growth factor alpha (TGF-α)	regulates the growth and differentiation of mesenchymal cells, endothelium, and epithelium; affects the formation, reconstruction, and regeneration of bones by inhibiting collagen synthesis and release of calcium; regulates osteogenesis by influencing the formation of osteoblasts and the deposition of bone matrix.	platelets, macrophages, keratinocytes	[12,13]
transformative growth factor beta (TGF-β1)	stimulates the chemotaxis of fibroblasts; increases the synthesis of type I collagen and fibronectin, and regulates the secretion of collagenase; stimulates or inhibits endothelial, fibroblastic, and osteoblastic mitogenesis; inhibits DNA synthesis in human fibroblasts; regulates the mitogenic action of other growth factors (*EGF*, *PDGF*, *aFGF*, *bFGF*); stimulates endothelial chemotaxis and angiogenesis; participates in the regulation of the balance between fibrosis and myocyte regeneration; inhibits the formation of osteoclasts and bone resorption; inhibits the proliferation of macrophages and lymphocytes.	platelets, T-lymphocytes, macrophages/monocytes, neutrophils, extracellular matrix of bone, cartilage, activated TH1 cells and natural killer cells	[6,7,9,10,11,12,13]
keratinocyte growth factor (KGF)	participates in wound healing; acts as a myogen/myocyte for many epithelial cells except endothelial cells and fibroblasts; increases collagen production by creating a support substance for the skin; is responsible for the growth and a new generation of keratinocytes.	platelets, fibroblast	[12,13,15,16]
acidic fibroblast growth factor (aFGF or FGF-1)	it is a myogen for skin keratinocytes, fibroblasts, and skin endothelial cells; stimulates the proliferation of myoblasts and mesenchymal cells; retards the growth and differentiation of chondrocytes and osteoblasts; stimulates the growth of epithelial cells; supporting wound healing.	platelets, macrophages	[6,7,10,12,13,16]
basic fibroblast growth factor (b-FGF or FGF-2)	activates the production of KGF; regulates angiogenesis and wound contraction; promotes collagen synthesis, matrix, and epithelization; is responsible for the growth and differentiation of fibroblasts, myoblasts, osteoblasts, nerve cells, endothelial cells, keratinocytes, and chondrocytes; acts as a mitogen for mesenchymal stem cells; stimulates the proliferation of myoblasts.	platelets, macrophages, mesenchymal cells, chondrocytes, osteoblasts	[6,7,8,12,13]
vascular endothelial growth factor (VEGF/VEP)	induces neovascularization by promoting proliferation and migration of macrovascular endothelial cells; promotes angiogenesis, participates in the formation of blood vessel lumen (indirectly through the release of nitrous oxide); initiates the regeneration of blood circulation and supports wound healing; activates the synthesis of metalloproteinase, involved in the degradation of interstitial collagen types 1, 2 and 3; stimulates the chemotaxis of macrophages and neutrophils;	platelets, endothelial cells	[6,7,10,11,12,13]
connective tissue growth factor (CTGF)	stimulates the proliferation, migration and curling up of vascular endothelial cells; initiates osteoblast proliferation and differentiation and matrix mineralization; promotes angiogenesis, cartilage regeneration and platelet adhesion; participates in the tube formation of vascular endothelial cells.	platelets, fibroblasts, endothelial cells, chondrocytes, smooth muscle cells	[12,13,17]
granulocyte/macrophage colony-stimulating factor (GM-CDF or CSFa)	stimulates the proliferation and differentiation of osteoblasts, BM progenitor cells and fibroblasts; acts as a powerful chemoattractant for neutrophils; increases expression of selectin, integrins and adhesion molecules from the immunoglobulin superfamily; promotes angiogenesis; inhibits the proliferation and growth of keratinocytes, participates in burn reactions.	platelets, macrophages, T cells, mast cells, natural killer cells, endothelial cells and fibroblasts	[12,13]
tumor necrosis factor (TNF-α)	stimulates the growth of fibroblasts; promotes angiogenesis, inhibits the proliferation and growth of keratinocytes; participates in burn reactions.	platelets, inflammatory cells	[12,13]
insulin-like growth factor (IGF)	stimulates the growth of myoblasts and fibroblasts, activates the synthesis of collagenase and prostaglandin E2 in fibroblasts; regulates the metabolism of articular cartilage through increased synthesis of collagen and matrix osteon; stimulates cartilage growth, bone matrix formation and replication of preosteoblasts and osteoblasts; together with *PDGF* it can increase the speed and quality of wound healing by activating collagen synthesis; mediates the growth and repair of skeletal muscles;	platelets, osteoblasts, macrophages, monocytes, chondrocytes	[6,7,9,10,11,12,13]
interleukin 1β (IL-1β)	inhibits the growth of endothelial cells and hepatocytes; intensifies infectious processes and strongly stimulates fibroblasts; in high concentrations: activates osteoclasts, inhibits the formation of new bone, increases infection and collagenase activity; in low concentrations: supports the growth of new bone.	thrombin-activated platelets, cells of the innate immune system, such as monocytes and macrophages	[12,13,18,19]
interleukin 6 (IL-6)	stimulates the growth of fibroblasts and collagen production; together with IL-8, it promotes angiogenesis and is mitogenic for epithelial cells.	platelets, osteoblasts, mature fibroblasts, and macrophages	[13,19]
interleukin 8 (IL-8)	stimulates mitosis of epidermal cells; supports angiogenesis; induces chemotaxis in target cells, mainly neutrophils, but also other granulocytes, causing them to migrate toward the site of infection; stimulates phagocytosis at the target site.	platelets, macrophages, and other cell types such as epithelial cells, airway smooth muscle cells and endothelial cells, monocytes, neutrophils, and fibroblasts	[12,13,20]
platelet-derived epidermal growth factor(PDEGF)	stimulates endothelial angiogenesis; regulates the secretion of collagenase; stimulates epithelial and mesenchymal mitogenesis; supports wound healing by stimulating the proliferation of keratinocytes and dermal fibroblasts	platelets, macrophages, monocytes	[9,11]
platelet-derived angiogenesis factor (PDAF)	enhances angiogenesis and vascular permeability by stimulating vascular endothelial cells; promotes the mitogenesis of endothelial cells.	platelets, endothelial cells	[9,11]
platelet factor 4 (PF4)	play a role in wound repair and inflammation; stimulates the initial reflux of neutrophils into wounds; neutralizes of heparin-like molecules on the endothelial surface of blood vessels, inhibits local antithrombin activity and promotes coagulation.	alpha-granules of activated platelets	[9]

**Table 2:** Platelet-rich plasma therapy pros and cons [65,66,67].

Advantages	Disadvantages
Simple and minimally invasive technique (without the involvement of any surgery, incisions, or healing)	Injection site morbidity
Immediate preparation of PRP, which does not require any preservative facilities	Standardized method for the preparation and administration of PRP remains lacking
Safety of PRP preparations through use of own cells without any further modification	Scar tissue formation and calcification at the injection site
PRP therapy can restore both structure and function; intra-articular injections of PRP can simultaneously reduce synovial inflammation, protect cartilage, and reduce pain	Optimal processing time and isolation methods for platelets and leukocytes and the optimal concentration of these components for maximal beneficial effects remain unknown
Minimization of blood borne contaminants	Rare infections at the site of injury and allergic reactions
Shortening the recovery period involved in PRP	Optimal frequency and volume of PRP injections remain unknown
Biocompatibility and reduction the risk of the body rejecting or any other allergic reaction to the treatment; preparations do not elicit immune response	Contraindications for the supply in persons diagnosed with platelet dysfunction syndromes, thrombocytopenia, hyperfibrinogenemia, hemodynamic instability, sepsis, acute and chronic infections, chronic liver disease, anticoagulation therapy
